# The effects of ACSM-based exercise on breast cancer-related lymphoedema: a systematic review and meta-analysis

**DOI:** 10.3389/fphys.2024.1413764

**Published:** 2024-07-23

**Authors:** Biqing Luan, Zhiqiang Li, Qizhi Yang, Zhihui Xu, Yaqin Chen, Meiting Wang, Wenlin Chen, Fei Ge

**Affiliations:** ^1^ Department of Breast Surgery, First Affiliated Hospital of Kunming Medical University, Kunming, Yunnan, China; ^2^ The Third Department of Breast Surgery, Peking University Cancer Hospital Yunnan, Yunnan Cancer Hospital, The Third Affiliated Hospital of Kunming Medical University, Kunming, Yunnan, China

**Keywords:** breast cancer-related lymphedema, ACSM, exercise, systematic review, meta-analysis

## Abstract

**Background:**

Breast cancer-related lymphedema (BCRL) frequently occurs after axillary lymph node dissection and remains incurable even with lymphaticovenular anastomosis. Exercise interventions have emerged as a potential non-pharmacological management approach. However, standardized exercise recommendations tailored to BCRL patients are lacking.

**Purpose:**

This study evaluated the impact of high and low compliance exercise interventions, aligned with ACSM recommendations, on quality of life (QOL), shoulder range of motion (ROM), and arm volume in BCRL patients. It further aimed to determine the optimal exercise dosage, assessed via the FITT (frequency, intensity, time, type) principle, that maximizes health benefits for BCRL patients.

**Methods:**

Adhering to the PRISMA guidelines for systematic reviews and meta-analyses, we conducted a comprehensive literature search in various databases, including PubMed, Embase, Cochrane Library, and Web of Science, encompassing the period from the inception of these databases to December 2023. We extracted data on exercise form, frequency, intensity, duration, repetitions, and sets from the identified studies. Subsequently, a meta-analysis and review were conducted. The exercise interventions were evaluated based on ACSM recommendations and categorized as either high or low compliance with ACSM standards. Fixed or random effects models were employed to compare outcomes across study subgroups with comparable results. Additionally, funnel plot analyses, sensitivity analyses, and Egger’s and Begg’s tests were conducted to evaluate the potential for bias.

**Results:**

15 studies encompassing 863 patients with BCRL were analyzed. Eleven studies exhibited high ACSM compliance, while four demonstrated low ACSM compliance. Regarding QOL, the overall standard mean difference (SMD) was 0.13 (95% CI: −1.07, 1.33). Specifically, the SMD for the high-adherence subgroup was 0.91 (95% CI: 0.33, 1.49; *p* = 0.002). For ROM, the overall SMD was 1.21 (95% CI: −0.19, 2.61). For arm volume, the overall SMD was −0.06 (95% CI: −0.22, 0.10). QOL results differed significantly in the high-adherence subgroup, whereas no significant effect on ROM or arm volume was observed.

**Conclusion:**

The study revealed significant QOL improvements in patients with high ACSM compliance, contrasted with those with low compliance. Conversely, no notable changes in ROM or arm volume were observed. Notably, the high adherence group tended to show better ROM during exercise and stable arm volume. Future research is needed to validate these findings.

## 1 Introduction

Breast cancer, a heterogeneous disease influenced by genetic and environmental factors (Barzaman et al., 2020). Since the mid-2000s, the incidence of this malignancy has been gradually increasing by approximately 0.5% annually (Siegel et al., 2023). The lymphatic system plays a pivotal role in maintaining fluid homeostasis, nourishment, and immune defense. Lymphedema, a condition arising from obstructed lymphatic reflux, manifests as the accumulation of protein-rich interstitial fluid within subcutaneous tissues and skin. This accumulation results in swelling and skin alterations, which in severe instances can progress to cellulitis. Follow-up of patients after the end of treatment is easily overlooked, the recent decade has witnessed a surge in both understanding and therapeutic strategies for managing lymphedema in humans. Notably, cancer-related treatments, including extensive lymph node dissection, radiotherapy, and chemotherapy, are significant predisposing factors for the development of lymphedema (Bittar et al., 2020; Wong and Furniss, 2020; Rockson, 2021).

Breast cancer-related lymphoedema (BCRL) stands as one of the most serious complications arising from breast cancer. It is characterized by enlarged limb circumference, fibrotic tissue formation, inflammation, abnormal fat deposition, and ultimately, severe skin pathology, often accompanied by a heightened risk of recurrent skin infections. Notably, more than 20% of female breast cancer survivors will develop upper extremity lymphedema on sick side, and the occurrence of this condition is exacerbated within 2 years of breast cancer diagnosis or surgical intervention, and it is approximately fourfold more common in women who have undergone axillary lymph node dissection (Donahue et al., 2023). This debilitating condition often for the lifetime of the patient, posing a constant threat to nearly all breast cancer survivors. Its chronic nature significantly impacts the quality of life (QOL) and emotional wellbeing of affected individuals, while also imposing a substantial economic toll on patients, healthcare systems, and society at large (DiSipio et al., 2013). Currently, there are no approved medications specifically for treating lymphedema (Rockson et al., 2019). The primary treatment modalities consist of combination decongestive therapy (CDT), compression hosiery, photobiomodulation (PBM), surgical interventions, etc (Harmer, 2009; Storz et al., 2017). Surgical options primarily encompass lymphovascular-venous anastomosis (LVA), vascular lymph node transfer (VLNT), and suction-assisted protein lipectomy (SAPL), among others (Donahue et al., 2023). However, it is noteworthy that current medical treatments are unable to fully eradicate BCRL. Instead, we can solely aim to prevent or mitigate its clinical manifestations through various therapeutic measures. Consequently, there is an urgent need to explore practical palliative strategies.

Previously, breast cancer survivors were often advised to refrain from engaging in strenuous exercise of the affected upper extremity, due to concerns that such activities might exacerbate or trigger the development of lymphedema. Additionally, patients who have recently experienced tumor treatment-induced fatigue are further discouraged from initiating exercise, as it may further exacerbate their condition (Kim et al., 2020). More than two decades ago, researchers demonstrated the significance of muscle deformation in lymphatic propulsion and the concept of lymphatic formation and propulsion within actively contracting skeletal muscle was experimentally verified, revealing the efficacy of lymphatic propulsion through contracting muscles (Havas et al., 1997). Therefore, theoretical studies support the implementation of exercise interventions as a viable approach to mitigating the symptoms and progression of such disorders. Exercise has garnered increasing attention due to its beneficial impact on patients with BCRL (Marchica et al., 2021). Enhanced upper extremity exercise significantly improves upper extremity function, decreases pain intensity, decreases symptoms of arm disability, and does not increase other complications or adverse events compared to usual care in women with breast cancer at risk for BCRL or who have had BCRL after breast cancer surgery (Hasenoehrl et al., 2020a; Bruce et al., 2021; Wang et al., 2023). Therefore, patients with BCRL will benefit from exercise and is an accessible, feasible, cost-effective and important adjunct to alleviate the symptoms of BCRL, and exercise interventions can be used as a viable strategy to alleviate the symptoms of this disease and its progression.

The American College of Sports Medicine (ACSM) advocates the prescription of exercise programs tailored to individuals of all ages who appear to be in good health. This encompasses a comprehensive approach encompassing cardiovascular, resistance, flexibility, and neuromotor training. Furthermore, it facilitates the adoption of behavioral strategies that promote physical activity. The core components of the exercise prescription encompass the frequency, intensity, time, and type of exercise, commonly referred to as the FITT principle (Garber et al., 2011). For a considerable period, the ACSM exercise prescription has been deemed as the global gold standard for scientific fitness in the fields of sports medicine, sports science, and health and fitness (Ferguson, 2014). It provides personalized guidance for patients with cardiovascular, pulmonary, metabolic, or other health issues, enabling them to safely enjoy the benefits of scientific exercise. Consequently, this study employs the ACSM exercise guidelines as the criterion for evaluating optimal exercise dosage. A prior meta-analysis (Hasenoehrl et al., 2020a) examining the impact of resistance exercise on BCRL and limb strength in breast cancer survivors revealed significant reductions in bioimpedance spectroscopy (BIS) values and notable enhancements in muscle strength following resistance exercise. Consequently, the study concluded that resistance exercise did not have any systematic negative effects on BCRL. Basha, M. A. et al. (Basha et al., 2022) experimentally demonstrated the effectiveness of a novel exercise modality, virtual reality (VR) training, which proved to be superior to resistance training in managing BCRL. Both exercise modalities improved the physical functioning of BCRL patients and their QOL in distinct ways. Hayes, S. C. et al.’s findings (Hayes et al., 2022) further support the application of exercise guidelines encompassing aerobic and resistance exercise, as well as unsupervised exercise guided by symptomatic response, to individuals who have already developed or are at risk of developing cancer-related lymphedema. The exercise program recommended by the exercise prescription may improve BCRL to some extent while preventing BCRL.

There is a relative lack of current research on the dose of exercise during exercise intervention, resulting in disparate outcomes. Yet to be studied whether exercise interventions that adhere closely to the guidelines outlined by the ACSM exert a more favorable impact on BCRL patients compared to those with lower adherence rates. The objective of this systematic review is to contrast the effects of high compliance with ACSM guidelines against those of exercise interventions with low compliance in patients with BCRL.

## 2 Materials and methods

The systematic literature review process, inclusive of the screening of pertinent articles, adhered strictly to the PRISMA (Preferred Reporting Items for Systematic Reviews and Meta-Analyses) guidelines (Liberati et al., 2009). Furthermore, the study was duly registered on PROSPERO (CRD: 42024518159).

### 2.1 Search strategy

We conducted a thorough literature search across multiple electronic databases, utilizing subject terms such as “Exercise,” “Exercise Therapy,” “Resistance Training,” and “Breast Cancer Lymphedema,” along with their respective free-form variations. This search encompassed a timeframe from inception to December 2023 and encompassed databases including PubMed, Embase, Web of Science, and the Cochrane Library. The comprehensive search strategy, including specific terms and criteria, is outlined in Supplementary Material S1. Additionally, we examined the references of pertinent review articles and retrieved articles from complementary studies. In cases where further clarification or information was required, we contacted the respective study authors.

### 2.2 Eligibility criteria

Eligibility criteria were established using the PICO framework: a) Population: patients diagnosed with BCRL; b) Intervention: any form of exercise, including aerobics, resistance training, and stretching; c) Comparator: absence of exercise-related treatment or alternative treatments such as routine physical therapy, standard care, family education, and psychotherapy; d) Outcome: changes in QOL, shoulder range of motion (ROM), and arm volume following exercise intervention. Articles that met the following criteria for arm volume measurement were included in the study: the measurement of limb volume primarily relies on the water displacement method, wherein the hand and arm are submerged in water, and the displaced water volume is measured. This approach serves as the gold standard for quantifying arm swelling in patients with lymphedema or detecting edema and lymphedema. Alternatively, another method involves measuring the circumference at intervals from the wrist to the axilla, calculating the volume of each arm segment using circumference-to-volume formulas, and summing the volumes. Ultimately, the standardized mean difference (SMD) is employed to normalize the various outcome variables. The following studies were excluded: conference abstracts, review articles, animal experiments, guidelines, and editorials; studies where the control group received any exercise; studies focusing on specific medications during exercise interventions; studies with unextractable data; and studies lacking a control group or non-randomized controlled trials (non-RCTs).

To mitigate bias in the literature review, two authors independently screened the titles and abstracts of retrieved articles for compliance with the inclusion criteria. If a study appeared to meet the criteria, the full text was retrieved. Subsequently, both authors independently evaluated the full text against the inclusion criteria. In cases of disagreement, a third author intervened and discussions were held until a consensus was reached. Finally, the adequacy and suitability of the outcome assessments and results presentation of all included studies were examined for meta-analysis purposes.

### 2.3 Data synthesis and analysis

Data extraction was independently conducted by two authors, BQL and ZQL. The primary outcomes analyzed in this study encompassed QOL, shoulder mobility, and arm volume. Tables were specifically designed to extract pertinent data, encompassing publication characteristics (including the first author’s name, year of publication, and country of origin), methodological attributes (such as the number of study groups, group design, interventions, and sample size), participant demographics (age, sex ratio, and lymphedema stage), and exercise parameters (form, frequency, intensity, duration, number of repetitions, and set numbers). Additionally, outcome characteristics were meticulously extracted. When encountering data presented in graphs or curves lacking clear textual descriptions, the Engauge Digitizer software was utilized for accurate data extraction. In cases where data were incomplete or ambiguous, efforts were made to obtain clarification from the respective study authors.

Following data extraction, exercise interventions were evaluated for compliance with the American College of Sports Medicine (ACSM) recommendations. These interventions were assessed based on the ACSM’s guidelines for enhancing cardiorespiratory fitness and upper extremity motor function in patients with BCRL. Both authors, QZY and ZHX, independently rated each aspect of the exercise intervention (encompassing frequency, intensity, duration, etc.) against the various ACSM-recommended criteria, thereby assessing the adherence to the prescribed exercise protocol. Each movement indicator was assigned a score ranging from 0 to 2, where a score of 2 represented compliance, a score of 1 denoted uncertainty or absence of reporting, and a score of 0 indicated non-compliance. In instances of disagreement between the two authors, a third author was consulted for further assessment and discussion, leading to a final consensus. Utilizing this scoring system, we determined the proportion of compliance with the ACSM-recommended exercise dose in each study. Studies with a compliance proportion of ≥75% were classified as exhibiting high compliance with ACSM recommendations, whereas those with a proportion of <75% were categorized as demonstrating low compliance (Table 1).

**TABLE 1:** Assessment of ACSM compliance.

Author, year	Cardiorespiratory exercise						Resistance exercise								Flexibility exercise						ACSM compliance	
	Frequency (days/week)		Intensity/workload		Duration (min)		Frequency (days/week)		Intensity/workload		Repetitions (times)		Sets (groups)		Frequency (days/week)		Intensity/workload		Duration (min)		Points	Percent
Do et al., (2015)							5	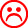	60% 1RM	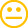	10	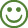	3	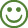							5/8	63%
Luz et al., (2018)							2	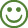	40% 1RM	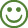	10→15	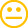	3	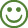							7/8	88%
Pasyar et al., (2019)															3	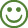	maximum stretch	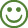	NR	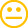	5/6	83%
Kilbreath et al., (2020)	3	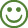	60%–85% of HRR	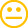	60	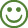	3	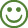	somewhat hard to extremely hard	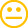	10∼12→14	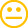	NR	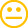							10/14	71%
Kizil et al., (2018)															7	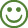	90% ROM	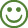	NR	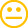	5/6	83%
Park, (2016)	5	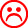	NR	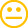	60	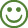	3	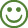	moderate to severe	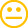	10	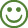	3	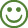							10/14	71%
Cormie et al., (2013)							2	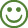	75%–85% 1 RM	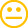	6∼10	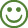	1∼4	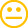							6/8	75%
Boeer et al., (2023)	1	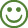	NR	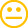	90	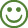									1	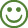	NR	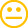	NR	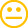	9/12	75%
Kim et al., (2010)							5	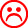	0.5-kg dumbbell→1-kg dumbbell	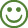	10	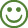	2	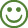							6/8	75%
Schmitz et al., (2009)							2	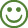	NR	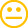	10	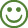	2→3	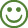							7/8	88%
Zhang et al., (2017)							2	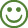	NR	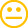	10	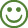	3	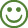							7/8	88%
Fong et al., (2014)															1	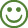	NR	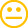	0.33	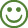	5/6	83%
McKenzie and kalda, (2003)	3	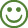	NR	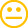	25	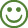	3	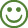	NR	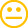	10	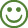	2→3	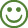							12/14	86%
Johansson et al., (2013)	3	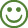	moderate	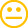	30	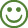	ct								3	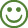	NR	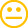	NR	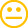	9/12	75%
Loudon et al., (2014)															1	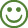	NR	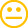	NR	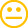	4/6	67%

Happy/green face, fulfills recommendation (2 points); Neutral/yellow face, uncertain fulfillment (1 point); Unhappy/red face, does not fulfill recommendation (0 points); ACSM, american college of sports medicine; HRR, heart rate reserve; 1 RM, one repetition maximum; NR, not reported.

### 2.4 Statistical analysis

To compare the results of the included studies, meta-analyses were conducted. Given the heterogeneity in scales and indicators used to assess continuous outcomes across studies, we employed standardized mean differences (SMDs) derived from random-effects models to estimate the combined intervention treatment effect size. Using Review Manager 5.4, we performed a meta-analysis to calculate SMDs, grouping the studies based on their level of adherence to ACSM recommendations. To assess statistical heterogeneity among the studies, we utilized the chi-square test and the Higgins I^2^ value. If the I^2^ value was below 50%, indicating minimal inconsistency across individual trials, a fixed-effects model was used to pool the results; otherwise, a random-effects model was applied. A statistically significant difference was considered present when *p* < 0.05. Additionally, funnel plots were generated to evaluate the potential for publication bias, with Begg’s rank correlation and Egger’s linear regression tests employed to assess the asymmetry of these plots.

### 2.5 Quality assessment

The quality of the included studies was evaluated by four authors—FG, WLC, YQC, and MTW—utilizing the quality assessment criteria for randomized controlled trials advocated by the Cochrane Collaboration (Higgins et al., 2011). The study included an assessment of various biases, specifically: random sequence generation (addressing selection bias), allocation concealment (addressing performance bias), blinding of participants and personnel (addressing performance bias), blinding of outcome assessment (addressing detection bias), incomplete outcome data (addressing attrition bias), selective reporting (addressing reporting bias), and other biases. According to the Cochrane Handbook, the risk of bias for each domain was categorized into three levels: “low risk,” “uncertain risk,” and “high risk.” The overall risk of bias is considered low if all domains are evaluated as posing a low risk of bias. If some domains are evaluated as posing an “uncertain risk” and none are categorized as high risk, the overall risk of bias is classified as “uncertain risk.” However, if the risk of bias for any single domain is evaluated as “high risk,” the overall risk of bias is designated as “high risk” (Cumpston et al., 2019).

## 3 Results

### 3.1 Literature search

**FIGURE 1:**
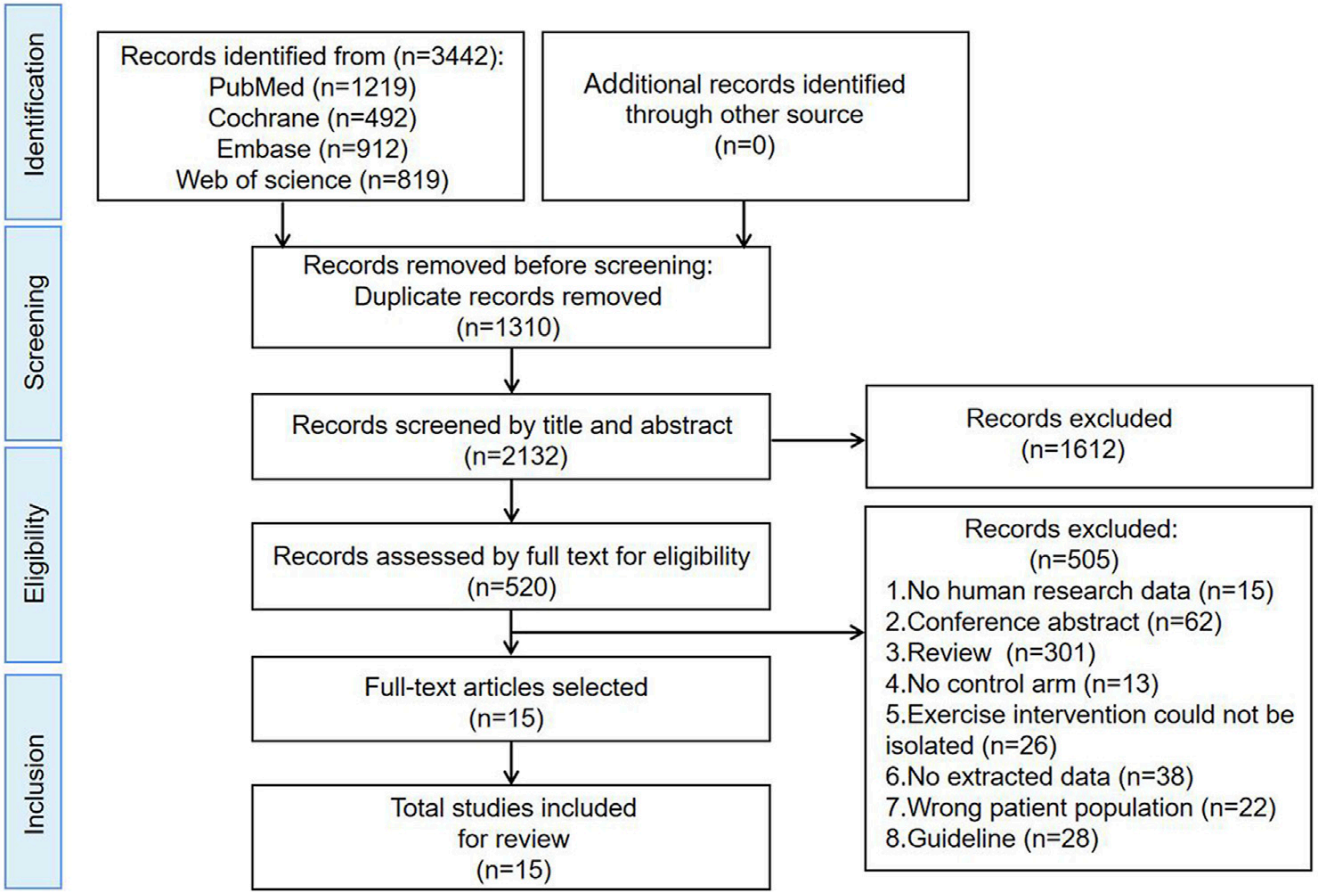
Search and selection procedures of the literature for the systematic review, described in detail by the Preferred Reporting Items for Systematic Reviews and Meta-Analyses (PRISMA) flowchart.

A comprehensive database search yielded a total of 3,442 literature. Out of these, 1,310 duplicate studies were promptly excluded, narrowing down the selection to 2,132 papers for initial evaluation. Upon meticulous review of their titles and abstracts, 1,612 articles were excluded due to their failure to fulfill the established inclusion criteria. Subsequently, the remaining 520 literature were read in full, resulting in the exclusion of various categories: 15 studies lacking human data, 62 conference abstracts, 301 reviews, 13 studies without a control group, 26 studies with exercise interventions that could not be isolated, 38 studies with no extractable data, 22 studies involving incorrect patient populations, and 28 guidelines. After a thorough re-evaluation of the full texts in comparison to the selection criteria, a final count of 15 studies was deemed eligible for inclusion (McKenzie and Kalda, 2003; Schmitz et al., 2009; Kim et al., 2010; Cormie et al., 2013; Johansson et al., 2013; Fong et al., 2014; Loudon et al., 2014; Do et al., 2015; Park, 2016; Zhang et al., 2017; Kizil et al., 2018; Luz et al., 2018; Pasyar et al., 2019; Kilbreath et al., 2020; Boeer et al., 2023). The PRISMA flowchart is given in Figure 1.

### 3.2 Study characteristics

The meta-analysis encompassed 15 studies spanning from 2003 to 2023, involving a total of 863 patients with BCRL. Specifically, 409 cases were assigned to the experimental group, while 454 cases comprised the control group. Notably, one study reported data from three separate arms (Cormie et al., 2013). After excluding a literature that did not provide the sex ratio (Kim et al., 2010), it was observed that only one male participant was included in the exercise intervention group of another study (Boeer et al., 2023). Remarkably, all participants in the remaining 13 studies were female, resulting in a near-100% female participation rate. The majority of patients in the included studies suffered from lymphedema grades I-III. However, two studies (Johansson et al., 2013; Luz et al., 2018) failed to report *p*-values for clinical characteristics. Furthermore, participants from one study (Kilbreath et al., 2020) exhibited significant age differences with a *p*-value of less than 0.01, while those from two other studies (Loudon et al., 2014; Boeer et al., 2023) displayed significant differences in body mass index characteristics, with *p*-values less than 0.05. For the remaining studies, clinical characteristics such as age, body mass index, education level, economic status, lymphedema grade, and lymphadenectomy did not differ significantly, with *p*-values exceeding 0.05. Geographically, the included studies encompassed a diverse range of countries, including Brazil (Luz et al., 2018), Iran (Pasyar et al., 2019), Turkey (Kizil et al., 2018), Germany (Boeer et al., 2023), China (Fong et al., 2014), Canada (McKenzie and Kalda, 2003), Sweden (Johansson et al., 2013), two United States (Schmitz et al., 2009; Zhang et al., 2017), three Australia (Cormie et al., 2013; Loudon et al., 2014; Kilbreath et al., 2020) and three from Korea (Kim et al., 2010; Do et al., 2015; Park, 2016) (Table 2).

**TABLE 2:** Basic characteristics of the study.

Author, year	Country	N		Age*		%Female		Lymphedema stage (%)	
		IG	CG	IG	CG	IG	CG	IG	CG
Do et al., (2015)	Korea	22	22	49.7 ± 7.05	49.6 ± 10.35	100%	100%	Unknown	Unknown
Luz et al., (2018)	Brazil	20	22	Unknown	Unknown	100%	100%	I:20.0 II:80.0	I:72.7 II:27.3
Pasyar et al., (2019)	Iran	20	20	51.6 ± 10.46	51.8 ± 11.4	100%	100%	0:5.0 I:10.0 II:75.0 III:10.0	0:10.0 I:20.0 II:60.0 III:10.0
Kilbreath et al., (2020)	Australia	41	47	53.7 ± 10.4	59.5 ± 8.0	100%	100%	Unknown	Unknown
Kizil et al., (2018)	Turkey	14	16	55.5 ± 8.3	58.0 ± 10.0	100%	100%	II/III:75.0	II/III:45.5
Park, (2016)	Korea	35	34	54.78 ± 3.42	52.48 ± 5.57	100%	100%	Unknown	Unknown
Cormie et al., (2013)	Australia	22	19	56.1 ± 8.1	58.6 ± 6.7	100%	100%	I:40.9 II:40.9 III:18.2	I:26.3 II:68.4 III:5.3
Boeer et al., (2023)	Germany	28	70	60.0 ± 9.2	62.5 ± 10.2	100%	100%	Unknown	Unknown
Kim et al., (2010)	Korea	20	20	50.50 ± 10.58	50.90 ± 9.15	Unknown	Unknown	Unknown	Unknown
Schmitz et al., (2009)	United States	71	70	56.0 ± 9.0	58.0 ± 10.0	100%	100%	0:7.0 I:25.0 II:45.0 III:23.0	0:10.0 I:17.0 II:37.0 III:36.0
Zhang et al., (2017)	United States	71	70	56.0 ± 10.0	58.0 ± 10.0	100%	100%	0:7.0 I:25.0 II:45.0 III:22.0	0:10.0 I:17.0 II:37.0 III:36.0
Fong et al., (2014)	China	11	12	58.3 ± 10.1	53.8 ± 4.2	100%	100%	Unknown	Unknown
McKenzie and kalda, (2003)	Canada	7	7	56.4 ± 10.4	56.9 ± 8.2	100%	100%	Unknown	Unknown
Johansson et al., (2013)	Sweden	15	14	64.0 ± 4.5	62.0 ± 3.3	100%	100%	I:20.0 II:47.0 III:33.0	I:21.0 II:57.0 III:21.0
Loudon et al., (2014)	Australia	12	11	55.1 ± 2.5	60.5 ± 3.6	100%	100%	Unknown	Unknown

*Mean ± SD; IG, intervention group; CG, control group.

Regarding the findings of these studies, 73% reported an exercise intervention lasting longer than 8 weeks. The frequency of these exercises spanned from once weekly to seven times weekly. 10 studies (McKenzie and Kalda, 2003; Kim et al., 2010; Cormie et al., 2013; Loudon et al., 2014; Do et al., 2015; Park, 2016; Kizil et al., 2018; Pasyar et al., 2019; Kilbreath et al., 2020; Boeer et al., 2023) reported on QOL, encompassing 487 patients with BCRL, with 221 patients in the intervention group and 266 in the control group. 5 studies (Cormie et al., 2013; Johansson et al., 2013; Park, 2016; Kizil et al., 2018; Luz et al., 2018) analyzed ROM, involving 211 patients with BCRL, split into 106 in the intervention group and 105 in the control group. 12 studies (McKenzie and Kalda, 2003; Schmitz et al., 2009; Kim et al., 2010; Cormie et al., 2013; Johansson et al., 2013; Fong et al., 2014; Loudon et al., 2014; Do et al., 2015; Zhang et al., 2017; Kizil et al., 2018; Luz et al., 2018; Pasyar et al., 2019) reported on arm volumes of participants, encompassing 628 patients with BCRL, with 305 patients in the intervention group and 303 in the control group. The remaining studies did not analyze these indices or the data were unextractable (Table 3).

**TABLE 3:** Study intervention and outcome reporting characteristics.

Author, year	Country	Interventions	Length of intervention	Outcomes		
				QOL	ROM	Arm volume
Do et al., (2015)	Korea	Resistance exercises	8 weeks	★		★
Luz et al., (2018)	Brazil	Strengthening Exercises	8 weeks		★	★
Pasyar et al., (2019)	Iran	Yoga exercise	8 weeks	★		★
Kilbreath et al., (2020)	Australia	Aerobic and resistance training program	12 weeks	★		
Kizil et al., (2018)	Turkey	Continuous passive motion	15 sessions	★	★	★
Park, (2016)	Korea	Aerobic exercise and strength training	4 weeks	☆	★	
Cormie et al., (2013)	Australia	High load resistance exercise	3 month	★	★	★
Boeer et al., (2023)	Germany	Paddling in a dragon boat	Unknown	★		
Kim et al., (2010)	Korea	Resistive exercise	8 weeks	★		★
Schmitz et al., (2009)	United States	Weight lifting	52 weeks			★
Zhang et al., (2017)	United States	Weight lifting	52 weeks			★
Fong et al., (2014)	China	Qigong exercise	6 min			★
McKenzie and kalda, (2003)	Canada	Upper Extremity Aerobic and resistance training	8 weeks	☆		★
Johansson et al., (2013)	Sweden	Water-Based Exercise	8 weeks		★	★
Loudon et al., (2014)	Australia	Yoga exercise	8 weeks	☆		★

★ The results were evaluated in the literature; ☆ Resulting data not extractable or sufficient for analysis; BIS, bioimpedance spectroscopy; QOL, quality of life; ROM, shoulder range of motion.

All studies incorporated distinct exercise interventions, such as resistance and strengthening exercises, yoga, dragon boat paddling, and water-based activities, among others. Similarly, each study employed a control group that encompassed various methods, including routine lymphedema care, rest, standard physical therapy, family education, and psychotherapy. Among the 15 studies examined, five (McKenzie and Kalda, 2003; Johansson et al., 2013; Park, 2016; Kilbreath et al., 2020; Boeer et al., 2023) specifically incorporated aerobic exercise, nine (McKenzie and Kalda, 2003; Schmitz et al., 2009; Kim et al., 2010; Cormie et al., 2013; Do et al., 2015; Park, 2016; Zhang et al., 2017; Luz et al., 2018; Kilbreath et al., 2020) focused on resistance training, and six (Johansson et al., 2013; Fong et al., 2014; Loudon et al., 2014; Kizil et al., 2018; Pasyar et al., 2019; Boeer et al., 2023) implemented stretching exercises, dragon boat rowing, yoga, and tai chi, as per the recommendations of the ACSM (Table 1).

### 3.3 Risk of bias

**FIGURE 2:**
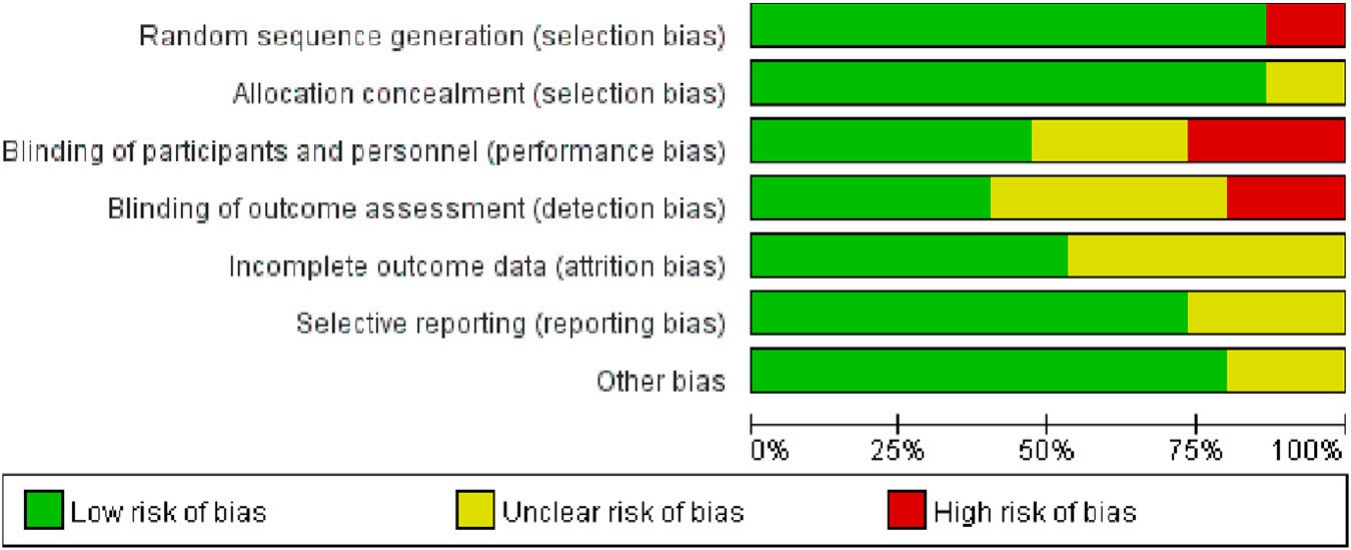
Combined percentage risk of bias in each risk domain for all included trials.

**FIGURE 3:**
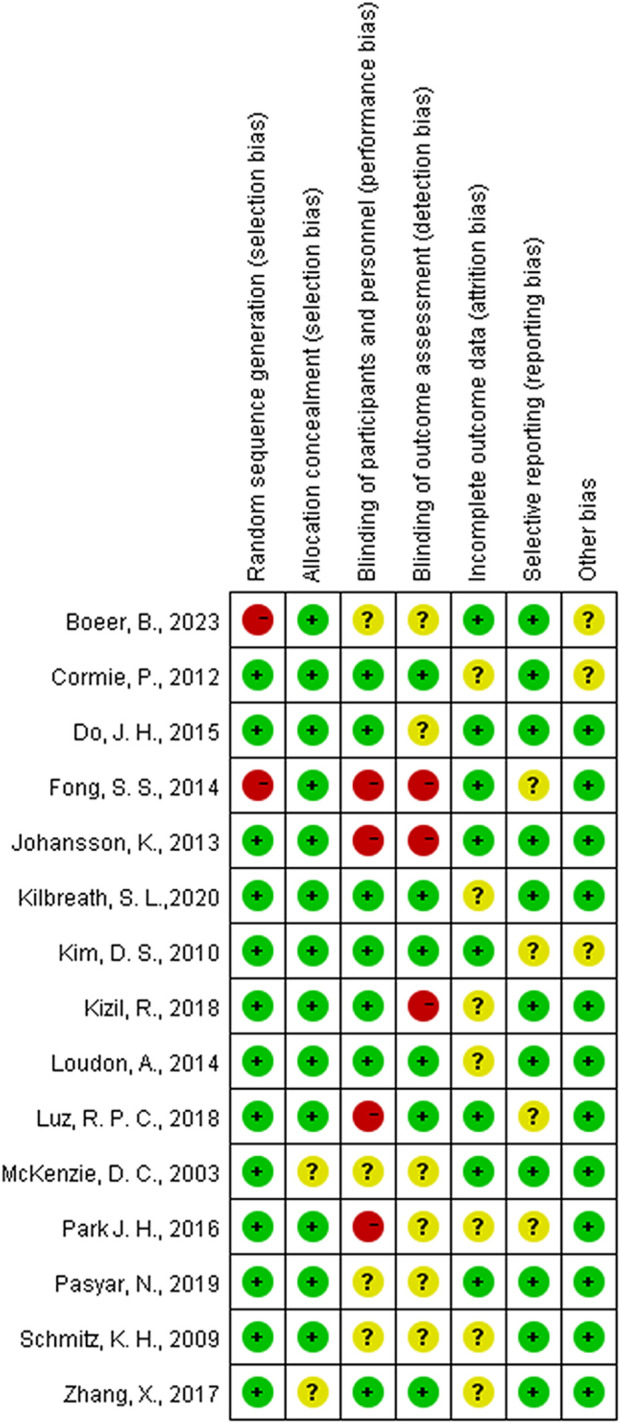
Risk of bias summaries for all exercise trials.

Among the 15 studies examined, 13 exhibited a low risk of bias in random sequence generation, while two studies, due to nonrandom assignment, were deemed to have a high risk. The absence of double-blinding emerged as the primary source of potential methodological bias. Given the challenges in implementing double-blinding in exercise interventions, the overall bias risk in this regard remains relatively elevated. Less than half of the studies reviewed reported blinding of participants and outcome assessors. Five studies were either single-blind or lacked blinding measures, thereby posing a high risk of bias. While five studies reported patient attrition, the number of lost patients was limited to ≤5, resulting in an indeterminate risk classification. The remaining 10 studies exhibited a low risk, as the number of subjects post-intervention closely resembled the baseline count. The risk of selective reporting bias was generally low, with only four studies raising concerns due to the omission of pre-registration program details. Three studies presented an uncertain risk of other biases (Figures 2, 3).

### 3.4 Compliance with the ACSM recommendations

We employed a specialized scoring system to assess compliance with ACSM guidelines. The intervention groups in the study were stratified into two categories based on the exercise dose: high compliance and low compliance. Eleven of the 15 studies (McKenzie and Kalda, 2003; Schmitz et al., 2009; Kim et al., 2010; Cormie et al., 2013; Johansson et al., 2013; Fong et al., 2014; Zhang et al., 2017; Kizil et al., 2018; Luz et al., 2018; Pasyar et al., 2019; Boeer et al., 2023) demonstrated adherence to ACSM recommendations for exercise interventions at ≥75%, whereas four studies (Loudon et al., 2014; Do et al., 2015; Park, 2016; Kilbreath et al., 2020) fell below this threshold. The low adherence observed in these studies was attributed to an experimental design that overlooked crucial aspects of the recommended prescription or insufficient information on the exercise prescription for a comprehensive evaluation (Table 1). Among the 10 studies utilizing QOL as an outcome measure, six exhibited high ACSM adherence, while four displayed low adherence. Notably, three of these studies had results that were not extractable or suitable for meta-analysis. Of the five studies employing ROM as an outcome metric, four demonstrated high ACSM adherence, with one study exhibiting low adherence. Furthermore, among the 12 studies that used arm volume as an outcome indicator, 10 studies showed higher ACSM adherence, while two studies exhibited lower adherence.

### 3.5 Meta-analysis

**FIGURE 4:**
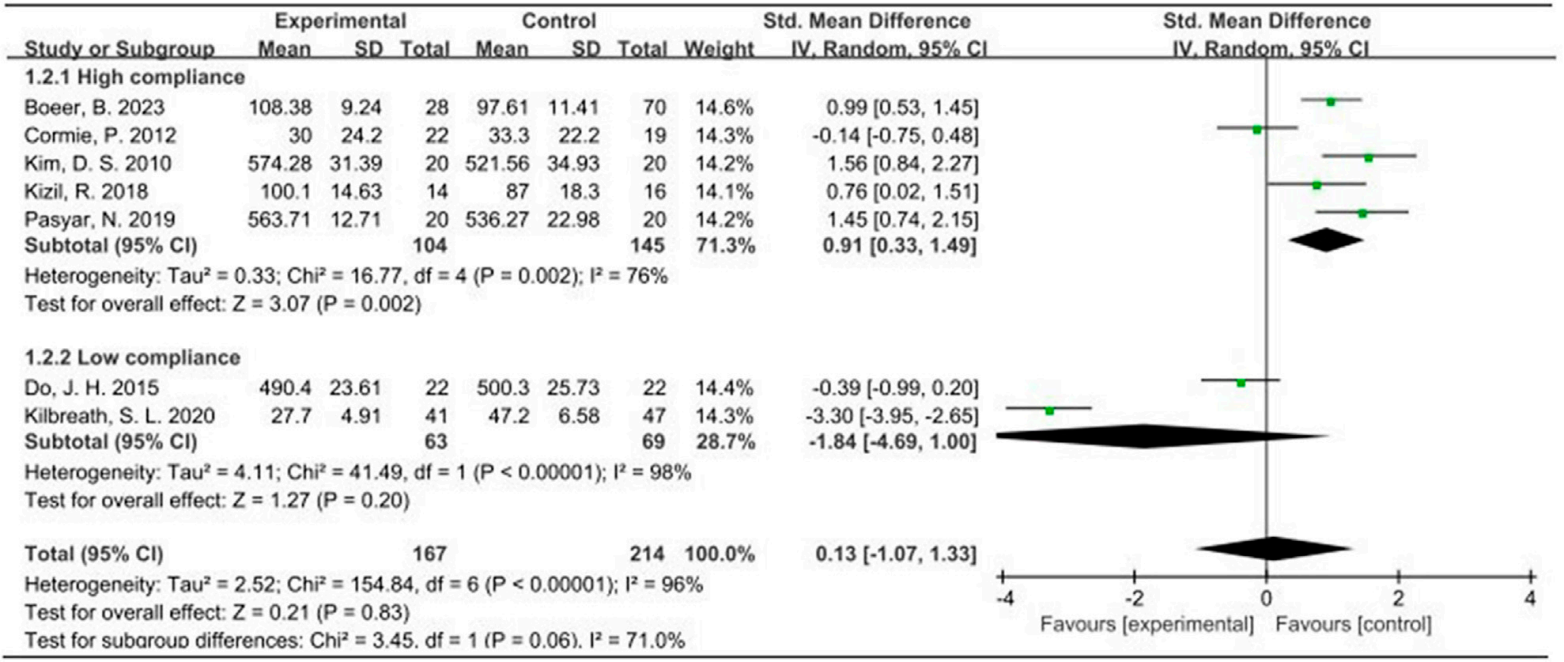
Forest plot of meta-analysis on the effect of exercise on QOL in BCRL patients.

**FIGURE 5:**
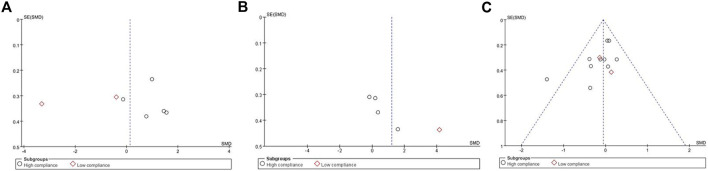
Funnel plot of meta-analysis on the effect of exercise on QOL, ROM and arm volume in BCRL patients.

Seven studies, encompassing 487 participants and utilizing QOL as the primary outcome measure, were analyzed using a random effects model due to an I^2^ value exceeding 50%. The pooled standardized mean difference (SMD) for QOL was 0.13, with a 95% confidence interval (CI) ranging from −1.07 to 1.33. Among the subgroup displaying high ACSM adherence, the SMD was 0.91 (95% CI: 0.33, 1.49), indicating a significant difference (*p* = 0.002). Conversely, the subgroup with low ACSM adherence exhibited an SMD of −1.84 (95% CI: −4.69, 1.00). Therefore, a notable difference in QOL was observed among those with high adherence to ACSM guidelines. Specifically, the exercise intervention group adhering strictly to ACSM recommendations outperformed the low adherence subgroup in terms of QOL (Figure 4). Visual inspection of the funnel plot revealed symmetry, indicating the absence of publication bias (Figure 5A). This conclusion was further supported by Begg’s test (*P* = 0.652) and Egger’s test (*P* = 0.872), both indicating no significant publication bias.

**FIGURE 6:**
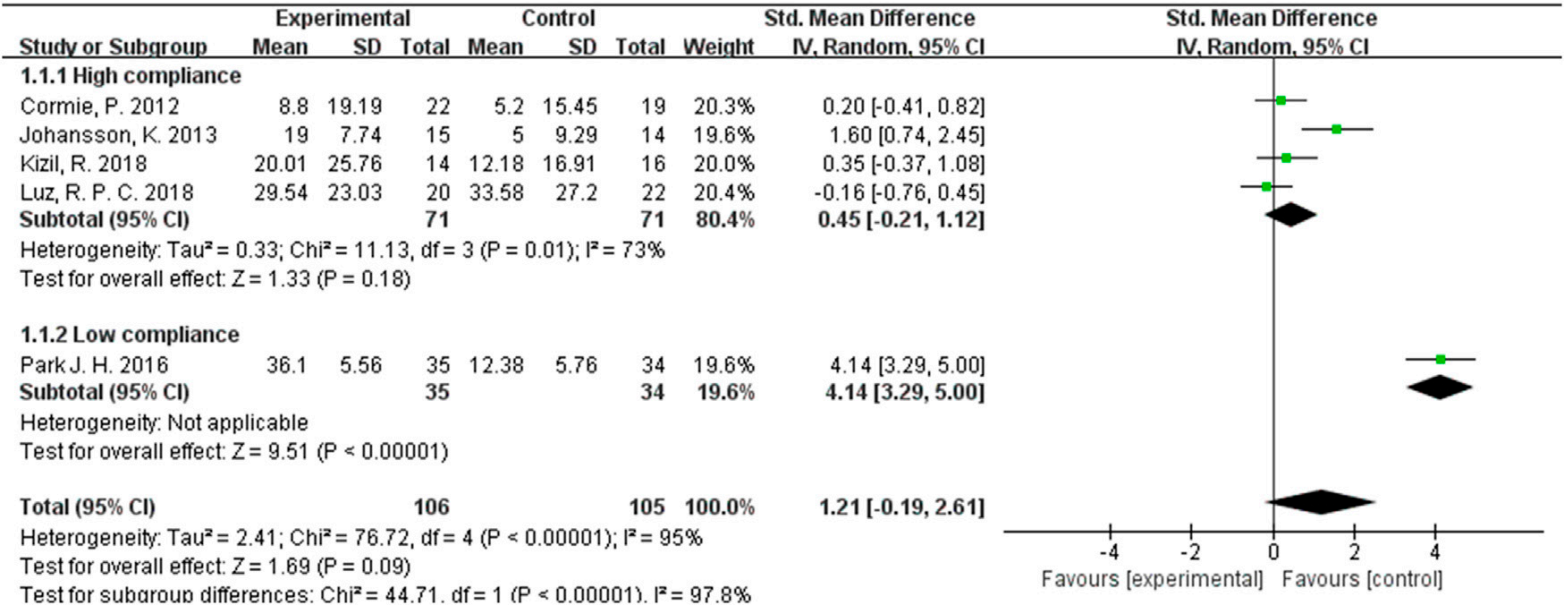
Forest plot of meta-analysis on the effect of exercise on ROM in BCRL patients.

In our analysis of ROM test results, we incorporated data from a total of 211 participants across five studies, exhibiting an I^2^ value exceeding 50%. These studies were evaluated using a random-effects model. The pooled standardized mean difference (SMD) for ROM was calculated as 1.21, with a 95% CI ranging from −0.19 to 2.61. Within the subgroup exhibiting high ACSM adherence, the SMD was found to be 0.45 (95% CI: −0.21, 1.12). In contrast, the SMD for the ACSM low adherence subgroup was 4.14 (95% CI: 3.29, 5.00). Consequently, no significant difference in ROM results was observed among those with high ACSM adherence (Figure 6). The group with high adherence also demonstrated an improved trend in ROM. Visual inspection of the funnel plot revealed symmetry, indicating the absence of publication bias (Figure 5B).

**FIGURE 7:**
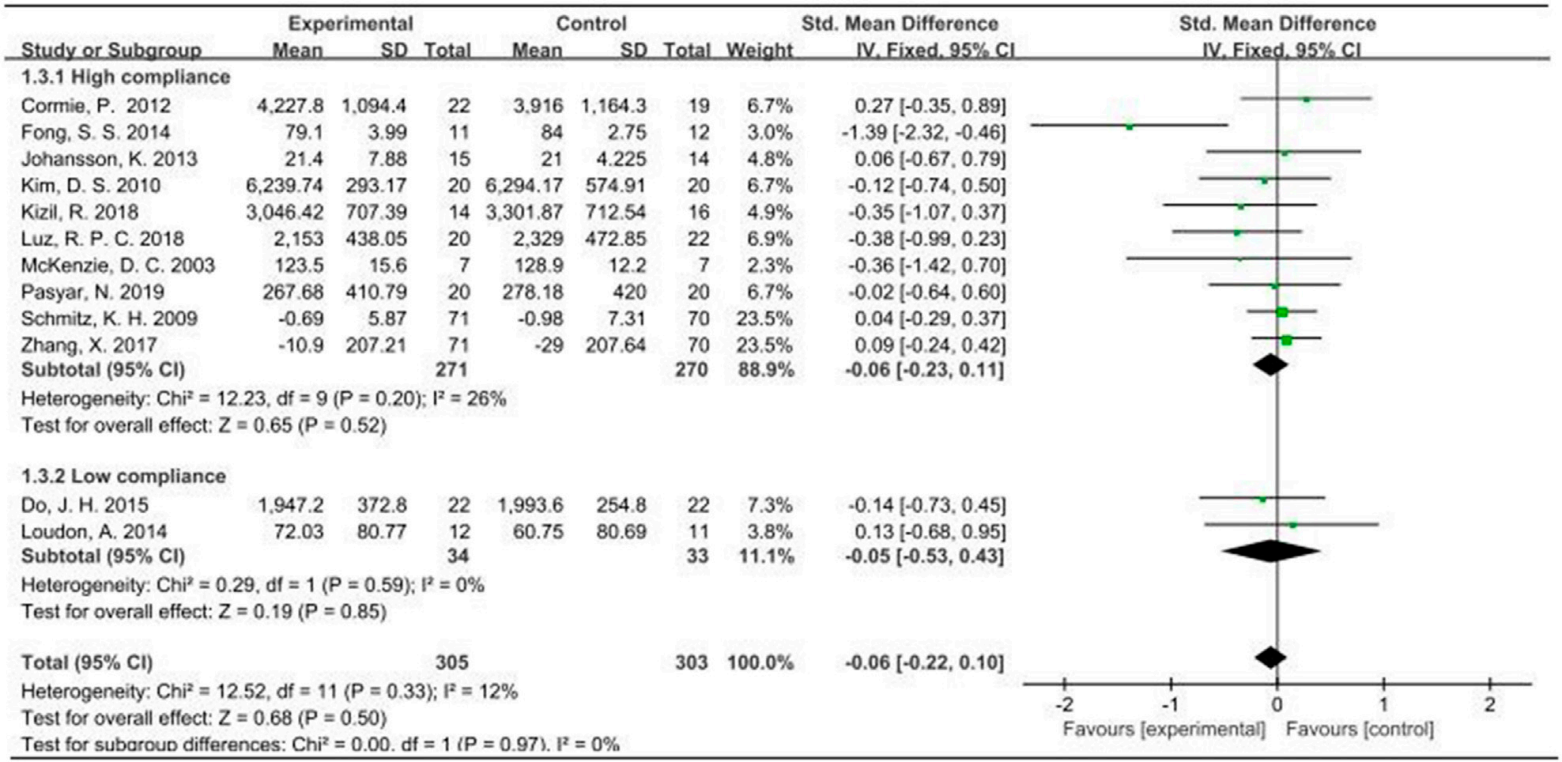
Forest plot of meta-analysis on the effect of exercise on arm volume in BCRL patients.

When the outcome was arm volume, a total of 628 participants from 12 studies were analyzed. Given that I^2^ was less than 50%, a fixed-effects model was employed for the analysis. The pooled standardized mean difference (SMD) for arm volume was −0.06, with a 95% CI ranging from −0.22 to 0.10. Notably, across the studies, the outcome measure of arm volume exhibited no heterogeneity among the two subgroups, as indicated by an I^2^ value of 0%. For the subgroup demonstrating high compliance with ACSM guidelines, the SMD was −0.06 (95% CI: −0.23, 0.11). Conversely, in the subgroup with low ACSM compliance, the SMD was −0.05 (95% CI: −0.53, 0.43). Similarly, no heterogeneity was observed in the low adherence subgroup for arm volume, with an I^2^ value of 0%. Overall, the results indicated no significant difference in arm volume between the high and low adherence subgroups to ACSM guidelines (Figure 7). However, it is noteworthy that the high adherence group did not demonstrate any worsening trend. The funnel plot exhibited symmetry, suggesting the absence of publication bias (Figure 5C).

**FIGURE 8:**
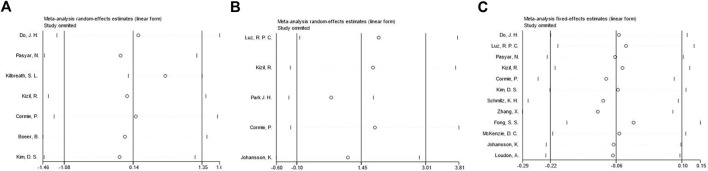
Sensitivity analysis of meta-analysis on the effect of exercise on QOL, ROM and arm volume in BCRL patients.

To investigate the sources of heterogeneity, we used sensitivity analysis. Based on the sensitivity analysis, we found that no individual study had a significant effect on the overall results, demonstrating the robustness of our findings (Figure 8).

## 4 Discussion

After conducting a thorough database search and iterative screening process, we successfully included 15 studies in our meta-analysis. These studies encompassed data on QOL, ROM, and arm volume measurements following exercise intervention among 863 BCRL patients from 10 distinct countries. Subsequently, we calculated compliance scores based on the ACSM-recommended exercise prescriptions, enabling us to compare and analyze the data within subgroups. When comparing the high adherence group (≥75% adherence) to the low adherence group (<75% adherence to ACSM recommendations), we observed QOL significantly improved in ACSM high adherence subgroups. Conversely, ROM and arm volume results exhibited no notable differences across the adherence subgroups. It is clear that high adherence to ACSM exercise prescription improves the overall quality of life of patients.

ACSM strives to facilitate the application of advancements in exercise science, physical education, and advanced science to human health through medical translation, thereby paving the way for improved well-being. ACSM establishes exercise dosages that aim to foster and sustain cardiorespiratory and muscular fitness, as well as flexibility, among healthy adults (American college of sports medicine position stand, 1998). Notably, adhering closely to ACSM exercise prescriptions can also confer benefits to individuals who are not in optimal health. Cui W, et al.’s (Cui et al., 2023) investigation into the impact of various exercise dosages on patients with Parkinson’s disease demonstrated that exercise intervention that adheres closely to ACSM guidelines is proven to be more effective in enhancing motor function, improving mobility, and enhancing QOL among patients with Parkinson’s disease. Similarly, Guo X, et al.’s (Guo et al., 2022) study in patients with knee osteoarthritis revealed significant differences in stiffness measures among those who adhered closely to ACSM exercise guidelines. However, despite these positive findings, there has been no meta-analysis conducted on ACSM-based exercise interventions in patients with BCRL. Therefore, the present study marks the first attempt to explore this area and has yielded similarly encouraging results. We hypothesized that ACSM may hold significant benefits in facilitating the comprehensive recovery of breast cancer patients. Specifically, it may enhance axillary and shoulder joint flexibility, expedite the restoration of muscle strength in compromised small muscle groups, facilitate the physiological balance among various muscle groups, and mitigate resistance to lymphatic return.

The passive mechanical compression of lymphatic vessels, induced by rhythmic muscular contractions and various directional movements, significantly contributes to the flow of lymphatic fluid. The primary mechanism responsible for this phenomenon is the lymphatic vessels’ intrinsic pumping function. The impetus for this movement is provided by the collecting lymphatic smooth muscle layer, generating phasic contractions that propel the lymph through its cycle. These contractions compress the lumen, generating fluid movement, while the unidirectional secondary valve system ensures lymphatic reflux (Breslin, 2014; Scallan et al., 2016).

Previous meta-analyses (Singh et al., 2016; Hayes et al., 2022) have corroborated the application of aerobic and resistance exercise, in addition to symptom-response-guided unsupervised exercise, offers advantages to individuals who have or are predisposed to developing cancer-related lymphedema (CRL), surpassing the benefits of resistance exercise alone. Notably, the relative risk of developing CRL in the exercise group was 0.90 (95% CI 0.7–1.13), indicating a favorable trend. For patients already diagnosed with CRL, the exercise group experienced improvements in pain, upper extremity function and strength, lower extremity strength, fatigue, and quality of life post-intervention (SMD = 0.3–0.8; *P* < 0.05). Similarly, patients who have developed cancer-related lymphedema can safely engage in gradual and regular exercise without exacerbation of their condition or related symptoms. Prior studies by Hasenoehrl T, et al. (Hasenoehrl et al., 2020a; Hasenoehrl et al., 2020b) and Keilani M, et al. (Keilani et al., 2016) have evaluated the impact of resistance exercise on BCRL. Their findings indicate that resistance exercise poses no adverse effects on BCRL patients and does not increase the risk of lymphedema progression in the affected limb. Despite the absence of a cure for BCRL, resistance exercise can serve as an active treatment modality for those affected. Additionally, Maccarone MC, et al. (Maccarone et al., 2023) a investigated the therapeutic benefits of water-based exercise for limb lymphedema, revealing improvements in QOL, and pain in the affected limb. However, these studies primarily focused on a single mode of exercise, whereas resistance, aerobic, and flexibility training are the most commonly employed modalities. Our study incorporates multidimensional exercise interventions as recommended by the ACSM Exercise Prescription. A broader array of projects is recommended for future research, as they are deemed to possess greater value than studies focusing solely on a specific type of exercise.

Current physical interventions for the clinical prevention and treatment of BCRL encompass a range of modalities, including CDT, lymphatic drainage massage, and the use of appropriate compression garments. These interventions have been implemented in BCRL rehabilitation programs and are actively being investigated in clinical trials. An analysis of published and unregistered clinical trials across various national registries reveals a diversity of therapeutic approaches, yet their reported outcomes are strikingly similar. The primary outcomes of these interventions are often related to motor function, changes in arm volume, and the QOL among BCRL patients. Consequently, our study focused on similar outcome measures in this patient population. The majority of patients in our study population were lymphedema grades I-III. From the results of our subgroup analysis, exercise interventions with high ACSM adherence showed significant differences in QOL outcomes in BCRL patients compared to exercise interventions with low ACSM adherence. Furthermore, we observed the integration of exercise interventions with physiotherapy techniques, such as VR-assisted training (Basha et al., 2022) and activity-oriented proprioceptive antiedema therapy (TAPA) (Muñoz-Alcaraz et al., 2020). Experimental results indicate that the combination of exercise and physical therapy interventions often leads to greater effectiveness than traditional physical therapy alone (Kim et al., 2010; Park, 2016; Kizil et al., 2018). Therefore, a combination of exercise interventions and physical therapy should be recommended for BCRL patients with a diversity of treatment strategies.

This meta-analysis exhibits several notable strengths. Firstly, a rigorous and comprehensive search was conducted across multiple databases, encompassing all studies that adhered to our established inclusion criteria, strictly adhering to the PRISMA guidelines (Liberati et al., 2009). Secondly, the focus of this systematic review and meta-analysis lies in exploring the impact of a comprehensive, multidimensional exercise program on patients with BCRL. Thirdly, the ACSM-recommended exercise interventions, encompassing aerobic, resistance, and stretching exercises, with precise guidelines on the optimal exercise dosage, were meticulously analyzed. A synthesis of various exercise modalities, intensities, and durations utilized in prior studies was conducted to assess the effectiveness of exercise interventions with varying degrees of ACSM adherence in BCRL patients. Fourthly, to our knowledge, no other meta-analysis has grouped studies based on ACSM adherence to assess the influence of interventions with high or low adherence on BCRL patients. Finally, a crucial aspect of this study is the inclusion of a blank control group, which did not receive any exercise intervention, providing a solid foundation for our analysis.

Our study is not without limitations. Firstly, the detailed descriptions of exercise prescriptions in the interventions are paramount for establishing a reasonable range of scores for ACSM adherence. However, inconsistencies in the reported frequency, intensity, and duration of exercises across the included studies. Consequently, it remains challenging to compile universal criteria for determining optimal exercise interventions. Secondly, some studies either failed to report or inadequately reported the exercise intervention doses. Consequently, there is a possibility that exercise intervention doses may have been indistinctly categorized into the low adherence or uncertain adherence group, despite adhering closely to ACSM recommendations. Finally, every study inherently carries the risk of introducing bias, which may have minimal impact on the overall population outcomes yet introduces uncertainty in the assessment of the effectiveness of specific interventions. Notably, the overall bias in this review may be more closely associated with the blinding of interveners and participants.

While acknowledging certain limitations, this review holds significant implications for clinical practice. The combination of physical therapy and an intensive exercise program could be a viable treatment option for clinicians and rehabilitation therapists managing patients with BCRL. These patients face unique health challenges, stemming from the impact of cancer treatment on both their physical and mental health. According to the Exercise Guidelines for Cancer Survivors (Campbell et al., 2019) exercise training and testing are deemed safe for cancer survivors, and it is recommended that all survivors engage in an appropriate exercise program. At present, various international institutions have formulated exercise recommendations tailored to individuals with cancer, yet we have opted one of them, ACSM guide. Had we chosen recommendations from alternative organizations, such as Exercise and Sports Science Australia (ESSA) (Hayes et al., 2019), the outcomes might have varied accordingly. Notably, 10 studies examined only a single type of exercise, while five involved two types. Based on our scoring system, no study achieved the perfect score. Therefore, future BCRL studies should incorporate more multidimensional exercise experiments. The optimal exercise program for BCRL ought to adhere to the principles of early individualization in accordance with ACSM guidelines, encompassing a rational integration of aerobic, resistance, and flexibility exercise. Enhancing compliance with a tailored exercise prescription, encompassing cardiorespiratory exercise, resistance training, flexibility exercise (CRF exercise prescription). Similar to other clinical principles, the management of patients with BCRL demands a personalized and precise approach, which may serve as a promising avenue for future research in this domain.

## 5 Conclusion

Our meta-analysis demonstrates significant differences and improvements in QOL measures among BCRL patients with high adherence (≥75%) to ACSM recommendations, in line with the recognized benefits of exercise in enhancing limb function, quality of life, and potential survival. Notably, no significant alteration in ROM and arm volume was observed following the exercise intervention. Furthermore, the high adherence group exhibited a tendency towards improved ROM in correlation with their exercise regimen. Arm volume also did not show a worsening trend in the high adherence group. However, the scientific evidence remains insufficient to conclusively support or refute the superiority of multidimensional exercise interventions in providing symptomatic relief to BCRL patients, necessitating further validation in future studies. Therefore, we recommend that future research explore and investigate additional dimensions of appropriate exercise regimens for BCRL patients, accounting for individual differences in lymphedema stage, geographic location, patient compliance, and preferences.
