# Supplementary material for: The effects of ACSM-based exercise on breast cancer-related lymphoedema: a systematic review and meta-analysis
Source: Front Physiol. 2024 Jul 23;15:1413764. doi: 10.3389/fphys.2024.1413764 (PMC11300927; doi:10.3389/fphys.2024.1413764)
Supplement: Supplementary file 1 [file Table1.docx]

Supplementary Material

**Supplementary TABLE S1.** Search strategy on Pubmed.

| #1 | (((((((exercise[MeSH Terms]) OR (Circuit-Based Exercise[MeSH Terms])) OR (Plyometric Exercise[MeSH Terms])) OR (Exercise Therapy[MeSH Terms])) OR (Resistance Training[MeSH Terms])) OR (Muscle Stretching Exercises[MeSH Terms])) OR (High-Intensity Interval Training[MeSH Terms])) OR (sports[MeSH Terms]) |
| --- | --- |
| #2 | ((((((((((((((((((((((((((((((((((((((((((((((((((((((((((((((((((((((((((((((((((((((((((((((((((((((((((((((((((((((((((((((((((((((((((((((((((((Exercises)) OR (Physical Activity)) OR (Activities, Physical)) OR (Activity, Physical)) OR (Physical Activities)) OR (Exercise, Physical)) OR (Exercises, Physical)) OR (Physical Exercise)) OR (Physical Exercises)) OR (Acute Exercise)) OR (Acute Exercises)) OR (Exercise, Acute)) OR (Exercises, Acute)) OR (Exercise, Isometric)) OR (Exercises, Isometric)) OR (Exercises, Isometric)) OR (Isometric Exercise)) OR (Exercise, Aerobic)) OR (Aerobic Exercise)) OR (Aerobic Exercise)) OR (Exercises, Aerobic)) OR (Exercise Training)) OR (Exercise Trainings)) OR (Training, Exercise)) OR (Trainings, Exercise)) OR (Circuit Based Exercise)) OR (Circuit-Based Exercises)) OR (Exercise, Circuit-Based)) OR (Exercises, Circuit-Based)) OR (Circuit Training)) OR (Training, Circuit)) OR (Training, Circuit)) OR (Exercise, Plyometric)) OR (Exercises, Plyometric)) OR (Exercises, Plyometric)) OR (Plyometric Drill)) OR (Drill, Plyometric)) OR (Drills, Plyometric)) OR (Plyometric Drills)) OR (Plyometric Training)) OR (Plyometric Trainings)) OR (Training, Plyometric)) OR (Trainings, Plyometric)) OR (Stretch-Shortening Exercise)) OR (Exercise, Stretch-Shortening)) OR (Exercises, Stretch-Shortening)) OR (Stretch Shortening Exercise)) OR (Stretch-Shortening Exercises)) OR (Stretch-Shortening Cycle Exercise)) OR (Cycle Exercise, Stretch-Shortening)) OR (Cycle Exercises, Stretch-Shortening)) OR (Exercise, Stretch-Shortening Cycle)) OR (Exercises, Stretch-Shortening Cycle)) OR (Stretch Shortening Cycle Exercise)) OR (Stretch-Shortening Cycle Exercises)) OR (Stretch-Shortening Drill)) OR (Drill, Stretch-Shortening)) OR (Drill, Stretch-Shortening)) OR (Stretch Shortening Drill)) OR (Stretch-Shortening Drills)) OR (Remedial Exercise)) OR (Exercise, Remedial)) OR (Exercises, Remedial)) OR (Remedial Exercises)) OR (Therapy, Exercise)) OR (Exercise Therapies)) OR (Therapies, Exercise)) OR (Therapies, Exercise)) OR (Exercise, Rehabilitation)) OR (Exercises, Rehabilitation)) OR (Rehabilitation Exercises)) OR (Training, Resistance)) OR (Strength Training)) OR (Training, Strength))) OR (Weight-Lifting Strengthening Program)) OR (Strengthening Program, Weight-Lifting)) OR (Strengthening Programs, Weight-Lifting)) OR (Weight Lifting Strengthening Program)) OR (Weight Lifting Strengthening Programs)) OR (Weight-Lifting Exercise Program)) OR (Exercise Program, Weight-Lifting)) OR (Exercise Programs, Weight-Lifting)) OR (Weight Lifting Exercise Program)) OR (Weight-Lifting Exercise Programs)) OR (Weight-Bearing Strengthening Program)) OR (Strengthening Program, Weight-Bearing)) OR (Strengthening Programs, Weight-Bearing)) OR (Weight Bearing Strengthening Program)) OR (Weight-Bearing Strengthening Programs)) OR (Weight-Bearing Exercise Program)) OR (Exercise Program, Weight-Bearing)) OR (Exercise Programs, Weight-Bearing)) OR (Weight Bearing Exercise Program)) OR (Weight-Bearing Exercise Programs)) OR (Exercise, Muscle Stretching)) OR (Exercise, Muscle Stretching)) OR (Static Stretching)) OR (Stretching, Static)) OR (Active Stretching)) OR (Stretching, Active)) OR (Static-Active Stretching)) OR (Static Active Stretching)) OR (Stretching, Static-Active)) OR (Isometric Stretching)) OR (Stretching, Isometric)) OR (Ballistic Stretching)) OR (Stretching, Ballistic)) OR (Dynamic Stretching)) OR (Stretching, Dynamic)) OR (Proprioceptive Neuromuscular Facilitation (PNF) Stretching)) OR (PNF Stretching)) OR (PNF Stretchings)) OR (Stretching, PNF)) OR (PNF Stretching Exercise)) OR (Exercise, PNF Stretching)) OR (PNF Stretching Exercises)) OR (Stretching Exercise, PNF)) OR (Proprioceptive Neuromuscular Facilitation)) OR (Neuromuscular Facilitation, Proprioceptive)) OR (Proprioceptive Neuromuscular Facilitations)) OR (Passive Stretching)) OR (Stretching, Passive)) OR (Relaxed Stretching)) OR (Stretching, Relaxed)) OR (Static-Passive Stretching)) OR (Static Passive Stretching)) OR (Stretching, Static-Passive)) OR (High Intensity Interval Training)) OR (High-Intensity Interval Trainings)) OR (Interval Training, High-Intensity)) OR (Interval Trainings, High-Intensity)) OR (Training, High-Intensity Interval)) OR (Trainings, High-Intensity Interval)) OR (High-Intensity Intermittent Exercise)) OR (Exercise, High-Intensity Intermittent)) OR (Exercises, High-Intensity Intermittent)) OR (High-Intensity Intermittent Exercises)) OR (Sprint Interval Training)) OR (Sprint Interval Trainings)) OR (sport)) OR (Athletics)) OR (Athletic) |
| #3 | #1 OR #2 |
| #4 | (Lymphedema[MeSH Terms]) OR (Breast Cancer Lymphedema[MeSH Terms]) |
| #5 | (((((((((((((((((((((((((((((((((((((((((((((((((((((((((Lymphedemas)) OR (Milroy Disease)) OR (Early Onset Lymphedema)) OR (Early Onset Lymphedemas)) OR (Lymphedemas, Early Onset)) OR (Hereditary Lymphedema)) OR (Hereditary Lymphedemas)) OR (Lymphedema, Hereditary)) OR (Lymphedemas, Hereditary)) OR (Hereditary Lymphedema 1)) OR (Hereditary Lymphedema 1s)) OR (Lymphedema, Early-Onset)) OR (Early-Onset Lymphedema)) OR (Early-Onset Lymphedemas)) OR (Lymphedema, Early Onset)) OR (Lymphedemas, Early-Onset)) OR (Hereditary Lymphedema Type I)) OR (Milroy's Disease)) OR (Milroys Disease)) OR (Nonne-Milroy Disease)) OR (Nonne-Milroy Disease)) OR (Nonne-Milroy Lymphedema)) OR (Lymphedema, Nonne-Milroy)) OR (Nonne Milroy Lymphedema)) OR (Nonne-Milroy-Meige Disease)) OR (Nonne Milroy Meige Disease)) OR (Primary Congenital Lymphedema)) OR (Congenital Lymphedema, Primary)) OR (Congenital Lymphedemas, Primary)) OR (Lymphedema, Primary Congenital)) OR (Lymphedema, Primary Congenital)) OR (Primary Congenital Lymphedemas)) OR (Congenital Familial Lymphedema)) OR (Congenital Hereditary Lymphedema)) OR (Congenital Hereditary Lymphedemas)) OR (Hereditary Lymphedema, Congenital)) OR (Hereditary Lymphedemas, Congenital)) OR (Hereditary Lymphedemas, Congenital)) OR (Lymphedemas, Congenital Hereditary)) OR (Lymphedema, Hereditary, Ia)) OR (Breast Cancer Lymphedemas)) ) OR (Lymphedema, Breast Cancer)) OR (Breast Cancer Treatment-Related Lymphedema)) OR (Breast Cancer Treatment Related Lymphedema)) OR (Breast Cancer-Related Arm Lymphedema)) OR (Breast Cancer Related Arm Lymphedema)) OR (Breast Cancer Related Lymphedema)) OR (Postmastectomy Lymphedema)) OR (Lymphedema, Postmastectomy)) OR (Lymphedemas, Postmastectomy)) OR (Postmastectomy Lymphedemas)) OR (Post-mastectomy Lymphedema)) OR (Lymphedema, Post-mastectomy)) OR (Post mastectomy Lymphedema)) OR (Post-mastectomy Lymphedemas) |
| #6 | #4 OR #5 |
| #7 | #3 AND #6 |
